# Fine-tuning the indirect electrochemical reaction in redox-mediated flow batteries

**DOI:** 10.1039/d5ra08926c

**Published:** 2026-02-05

**Authors:** Tulsi M. Poudel, Daphne E. Poirier, Marybeth Hope T. Banda, Eylul Ergun, Daniel Rourke, Kayode O. Ojo, Ertan Agar, Maricris L. Mayes, Patrick J. Cappillino

**Affiliations:** a Department of Chemistry and Biochemistry, University of Massachusetts Dartmouth Dartmouth Massachusetts 02747 USA pcappillino@umassd.edu; b Department of Mechanical Engineering, Energy Engineering Graduate Program, University of Massachusetts Lowell Lowell Massachusetts 01854 USA

## Abstract

Redox-mediated flow batteries (RMFBs) are a promising, emerging energy storage technology and have the potential to drastically increase the capacity of conventional redox flow batteries (RFBs) while maintaining their architectural flexibility. In these systems, a solution-phase active material is pumped between the RFB cell stack and storage tanks and is responsible for direct charge/discharge of the battery system. This material acts as a redox mediator (RM) between the electrochemical apparatus and a solid active material (SAM), which remains in the storage tanks and comprises the capacity of the system. Characteristics of the indirect electrochemical reaction between RM and SAM, which occur in the storage tank, external to the RFB stack, have so far been inferred from conventional RFB performance metrics. Herein, we report a study of this heterogeneous process that is based on spectroscopic measurements, carried out on the active materials, rather than interpretation of distal electrode processes. This provides independent information on the SAM's state-of-charge, a critical property of RMFB performance that is typically not measured directly. Further, we demonstrate that the redox reaction between the RM and the SAM, which is required for efficient operation, may be tuned by hundreds of mV, or even completely inhibited, by altering the type and concentration of supporting ions in the electrolyte. Finally, we report a periodic-DFT investigation of the vibrational spectroscopy of the SAM, which lays the groundwork for a thermodynamic framework that will be used to characterize and optimize the indirect electrochemical reaction.

## Abstract 2

An indirect electrochemical reaction is made viable through the oxidation of (RM) VBH^2−/1−^ and reduction of (SAM) CoHCF (Co^II^Fe^III^) accompanied by selective potassium-ion intercalation into the CoHCF lattice.

## Graphical abstract

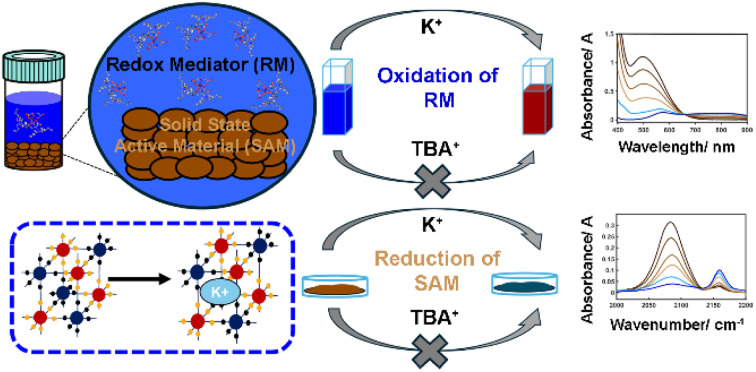

## 1 Introduction

According to the U.S. Energy Information Administration's Short-Term Energy Outlook, the coming years will see steady growth in electric power demand which, along with declining cost and government subsidies, will drive increasing demand in renewable energy technologies such as wind and solar.^1^ As such, battery capacity is required to mitigate intermittency inherent to these technologies, and replace fossil-fuel and pumped-hydropower reserve capacity.^1^ To meet this demand, redox flow batteries (RFBs) have been identified as a promising technology for long- and medium-duration storage.^2^ Technoeconomic analysis further suggests that this technology has the potential to meet current U.S. Department of Energy cost targets, especially if energy density can be improved.^3,4^

Despite the promise of RFBs in providing the storage necessary for a resilient, modern electrical grid, dependence of energy density on active-material solubility remains a critical challenge.^5–7^ It has been noted that, even when high-concentration can be achieved through electrolyte design,^8–11^ increased viscosity mitigates the benefits through pumping losses and in some systems this fundamentally limits energy density.^12^

**Fig. 1:**
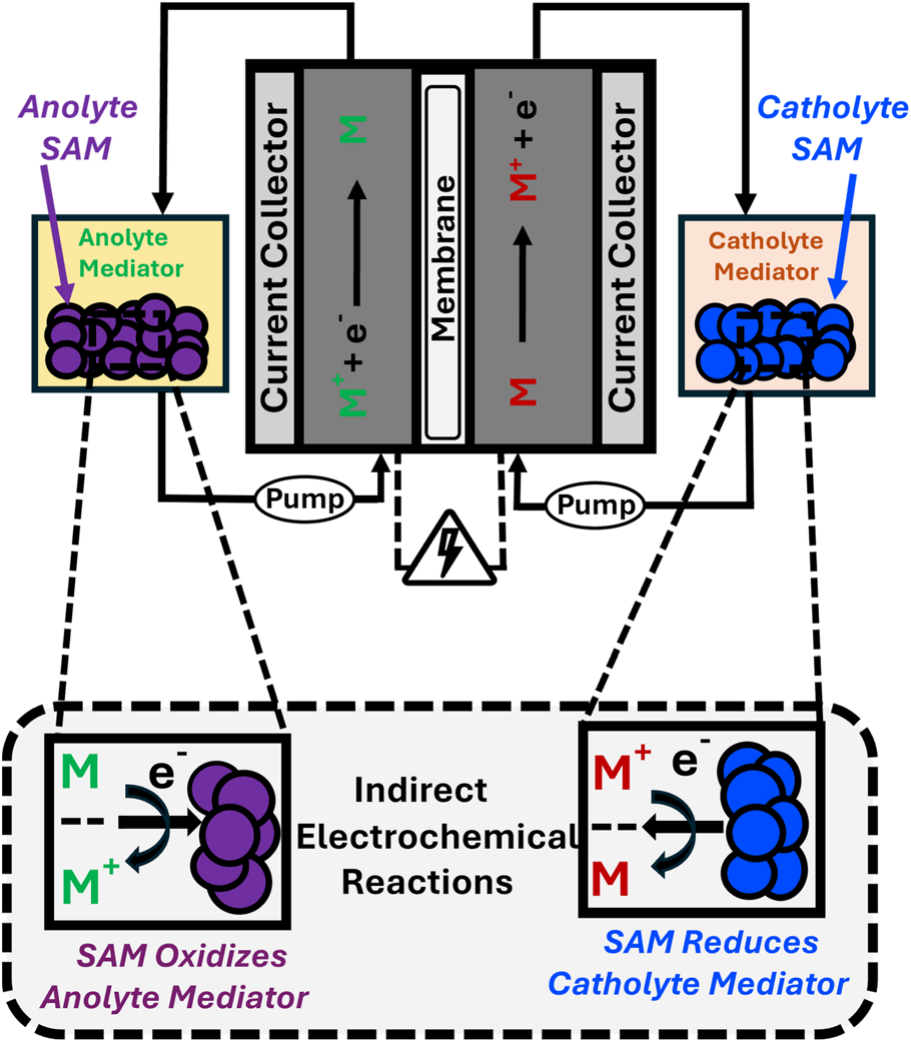
Schematic of a RMFB.

Redox-mediated flow batteries (RMFB) are a nascent technology with the potential to overcome challenge associated with RFBs, including low solubility of some active-materials and high viscosities associated with concentrated electrolytes.^13–19^ In these systems, a conventional RFB electrolyte is paired with a solid-state active material (SAM) which is stored in the electrolyte tanks (see Fig. 1). In this way, the solution-phase, RFB active-material acts as a redox mediator (RM) between the electrochemical cell and the SAM, which remains in the tanks. As the fraction of the total energy capacity shifts away from the RM and toward the SAM, these systems have the potential to approach the energy density of solid-state batteries. This facilitates lower concentration electrolytes flowing between the tanks and the electrochemical cell and preserves the principal advantage of RFBs over integrated batteries – decoupled storage capacity and power ouptut.^20^ Notably, at these lower-concentrations, compatible, solution-phase active materials may be mixed, providing a symmetric electrolyte that could be rebalanced,^8^ greatly reducing the operating requirements of membranes to reduce crossover and mitigating a key hurdle in RFB development.

With these potential benefits come trade-offs that must be balanced before this technology can be successfully deployed. Real world complexities of RMFBs currently under investigation^21–23^ include loss mechanisms, kinetic limitations, tank mixing inefficiencies and scalability concerns.

Successful implementation of RMFBs also comes with the fundamental technical challenge that – for a reversible cell, the charge/discharge (C/D) profile of the RM must be compatible with that of the SAM,^13^ such that:

• When oxidation of the RM is occurring in the electrochemical half-cell, its reduction potential must be greater than that of the SAM so that it may be spontaneously reduced in the tank.

• When reduction of the RM is occurring in the electrochemical half-cell, its reduction potential must be lower than the SAM, so that it may be spontaneously oxidized in the tank.

**Fig. 2:**
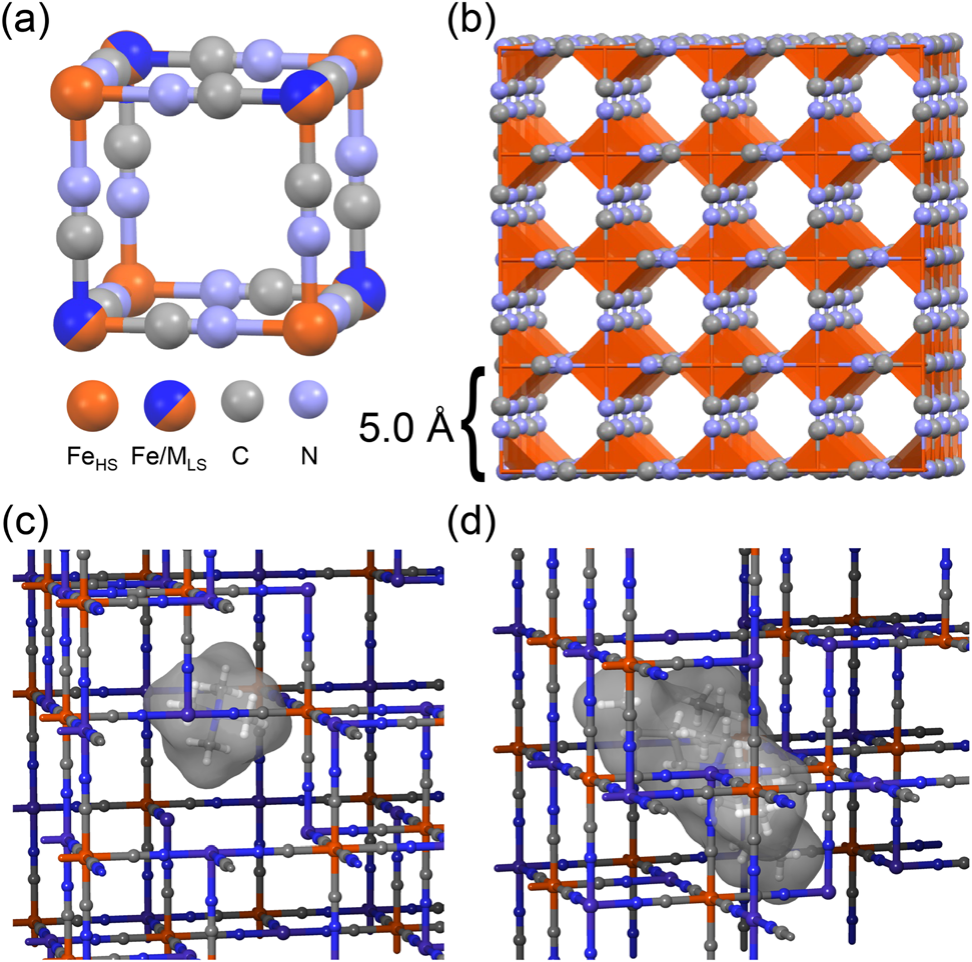
MHCM unit cell (a), lattice (b), the structures of tetramethylammonium (c) and tetrabutylammonium (d) intercalated within a MHCM lattice.

Several aspects of metal hexacyanometalates (MHCM), so-called Prussian Blue Analogs, which have been used in a number of seminal RMFB reports,^14,24–28^ suggest they are well-suited to address the significant challenge of redox matching. They have flat C/D curves, approaching ideal behavior.^29^ For example, a thermodynamic analysis of copper hexacyanoferrate demonstrated that 80% of its capacity (from 10–90% SOC) can be accessed with only a ∼150 mV potential sweep.^27^ Furthermore, this class of materials has been well-studied for energy storage applications, mostly in the context of metal-ion battery cathodes.^29–35^ It is extremely stable, even during redox cycling, and has very low solubility in most solvents. MHCMs are also diverse in composition because both the nitrogen-bound, high-spin metal ion and the carbon-bound, low-spin metal ion may be substituted in the lattice, which makes accessible a wide range of reduction potentials to pair with RMFB posolytes and negolytes (see Fig. 2).^36,37^

In this work we focus on a second critical aspect of MHCMs that makes them ideally suited for RMFB applications – the nature of the cations that intercalate/deintercalate within the MHCM lattice during reduction/oxidation alters its reduction potential in predictable ways.^38–41^ Through electrolyte design, the standard reduction potential and the slope of the charge/discharge curve of the SAM may be tuned to optimize its interaction with the RM. Furthermore, we demonstrate a strategy for directly and independently interrogating the states of charge of the SAM and the RM, by spectroscopically measuring the ratio of oxidized and reduced species. Because indirect, heterogeneous reactions between the SAM and the RM occur in the storage tanks, they are not subject to ohmic losses at electrodes. Therefore, characterizing these reactions chemically, supplemented by electrochemical measurements, will be crucial for a comprehensive understanding of RMFB operation. Herein, we report that an RM, vanadium bis-hydroxydiacetic acid (VBH)^8,42^ is stable in its reduced (V^4+^) state when mixed with an oxidized SAM (cobalt hexacyanoferrate, CoHCF), in the presence of bulky, tetrabutylammonium (TBA) cations, but that in the presence of smaller cations there is a large driving force for oxidation of VBH by CoHCF. Furthermore, we demonstrate that less bulky, tetramethylammonium (TMA) cations are capable of intercalating within the CoHCF, facilitating oxidation of VBH (see Fig. 2). Finally, these chemical redox investigations of the indirect electrochemical reaction between RM and SAM are corroborated using voltammetry of CoHCF films in the presence of electrolytes containing various cations.

## 2 Results and discussion

### 2.1 Chemical reduction of CoHCF by VBH electrolyte

The insertion process that occurs upon reduction of intercalation materials, whereby a cation occupies a discrete crystallographic lattice site, is well described in the literature.^39,41,43^ Despite this, the thermodynamic and kinetic details are not fully understood even for mature battery technologies that are widely commercially deployed, and remain the subjects of active investigation.^44,45^ This is especially the case in the context of MHCM, as these materials are most often studied as cathodes in the context of metal-ion batteries. Herein, we describe a strategy for chemical interrogation of the RMFB indirect electrochemical reaction (IER), which is a heterogeneous redox reaction that occurs in the electrolyte tank, between the RM (in this case VBH) and the SAM (in this case CoHCF). This reaction is completely decoupled from that occurring at the electrodes, which is conventionally used to characterize flow battery performance, and its direct measurement is an important step to better understanding RMFB operation.

**Fig. 3:**
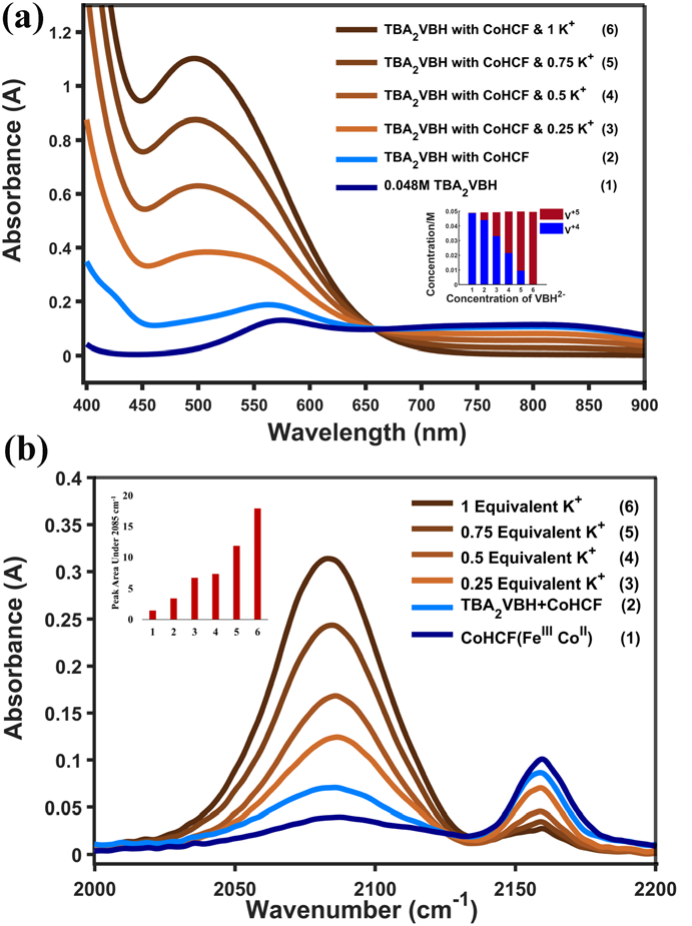
UV-vis spectra of VBH^2−/1−^, after addition of CoHCF and aliquots of between 0 and 1 equivalents of KPF6 (a) (inset: concentration of reduced (V^4+^) and oxidized (V^5+^) VBH). IR spectra of CoHCF for each sample shown in (b).

**Fig. 4:**
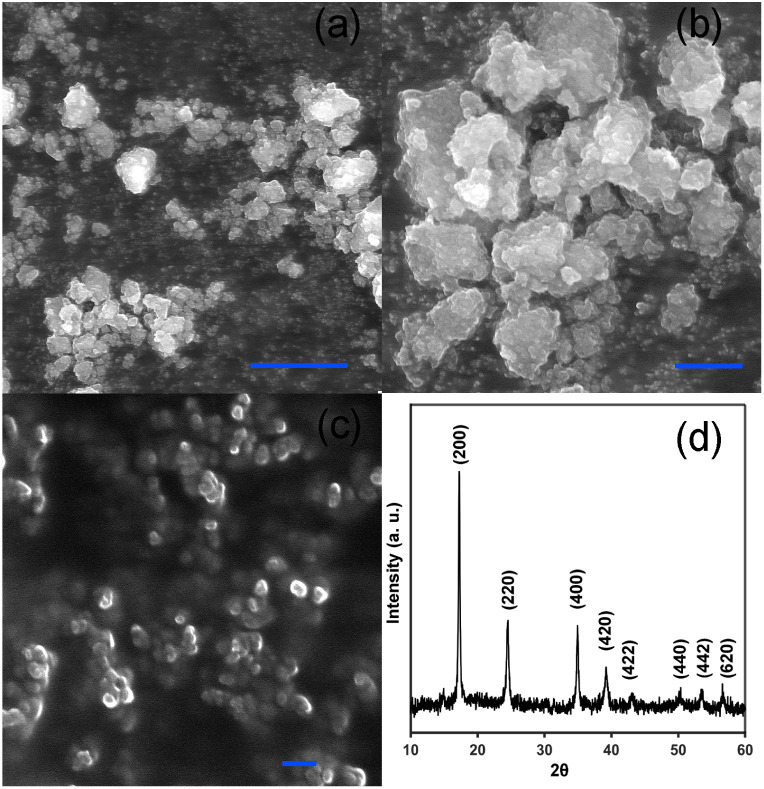
SEM images of CoHCF powder at 2 kX (a) 7 kX (b) and 35 kx (c) and powder XRD pattern (d). Scale bars in (a), (b), and (c) correspond to 10 µM, 2 µM and 200 nm, respectively.

Oxidation of solutions of VBH^2−^ (*E*° = 0.38 V *vs.* SHE in MeCN^42^) can be quantitated by simulating the UV-vis spectrum as a linear combination of authentic VBH^1−^ and VBH^2−^ authentic standards (see Fig. 3a).^9^ In these experiments, a full oxidizing equivalent of CoHCF was incubated with a solution of reduced VBH^2−^, in the presence of two equivalents of TBA^+^, with stirring, for one hour at room temperature, followed by centrifugation and spectral analysis. This results in ∼10% oxidation to VBH^1−^ (see Table 1), in line with what would be expected for surface adsorption of TBA cations, concomitant to reduction of the low-spin (C-bound) iron center. For example, considering spherical CoHCF particles with a diameter of 80 nm,^46^ ∼10% of the high-spin iron sites would be within 5 nm of the surface. This particle size in this example is consistent with SEM images of the CoHCF powders used herein, which are aggregates of 50–100 nm particles (see Fig. 4) and is also in line with literature reports of MHCMs prepared in a similar manner.^46,47^ The powder X-ray diffraction (XRD) pattern of CoHCF is also consistent with the pattern reported in literature.^47^ The steric bulk of the TBA cations preclude intercalation, limiting the reduction reaction to that which can be accommodated by adsorption of cations at the surface of CoHCF particles. This interpretation is consistent with the reported crystallographic radius of ∼0.494 nm for the TBA^48^ cation implied it is not well enough to fit inside the lattice of CoHCF framework.

**Table 1:** Percent oxidation of RM by SAM in the presence of TBA, TMA and K cations

mol TBA	mol TMA	mol K	mol CoHCF	% RM oxidation
6.3 × 10^−4^	—	—	3.3 × 10^−4^	11
9.4 × 10^−4^	—	—	3.3 × 10^−4^	9
6.3 × 10^−4^	3.3 × 10^−4^	—	3.3 × 10^−4^	18
6.3 × 10^−4^	—	3.3 × 10^−4^	3.3 × 10^−4^	100

In contrast, oxidation of VBH^2−^ with potassium ion insertion (ionic radius ∼0.138 nm^49^) into CoHCF (*E*° = 0.97 V *vs.* SHE^37^) is spontaneous by 0.59 V. Gradual addition of aliquots of KPF_6_, up to one full equivalent, cleanly affords quantitative oxidation of VBH^2−^ (see Fig. 3a and S1). Notably, this proceeds *via* addition of KPF_6_ only -no further redox reagents are added after the initial mixing of VBH^2−^ and CoHCF (Fig. 3a, light-blue trace). These results are consistent with recently reported quartz crystal microbalance investigations of MHCM thin films, which indicate redox intercalation of protons occurs even in the presence of TBA supporting salts but that the smaller cation, TMA (ionic radius ∼0.280 nm),^49^ impedes the process (see Fig. 2c).^50^

In corroboration of the controlled oxidation of VBH^2−^ by sequential addition of KPF_6_, concomitant, gradual reduction of CoHCF throughout the experiment can be observed by analyzing the solid CoHCF separated during centrifugation *via* infrared spectroscopy. The stretching frequency of cyanide (*ν*_CN_), near 2200 cm^−1^, provides a convenient means of characterizing the extent of CoHCF reduction. Due to σ-donation of electron density from Co and Fe-centers into the CN π* molecular orbital, this feature is sensitive to the oxidation state of the metals, shifting to lower frequency upon reduction of the metal ions.^51^Fig. 3b shows a shift of intensity from a peak at ∼2160 cm^−1^, matching that reported *ν*_CN_ of CoHCF(Fe^III^Co^II^), to near ∼2100 cm^−1^, matching that of CoHCF(Fe^II^Co^II^) after mixing VBH^2−^ with CoHCF.^51^ The peak ∼2100 cm^−1^ gradually increases throughout the course of KPF_6_, shifting quantitatively after addition of one full equivalent. These assignments are consistent with literature reports, both electrochemically deposited CoHCF^51–53^ and of films that were chemically deposited and on substrates^37,47^ as well as CV measurements of these CoHCF performed here in (*vide supra*).

**Fig. 5:**
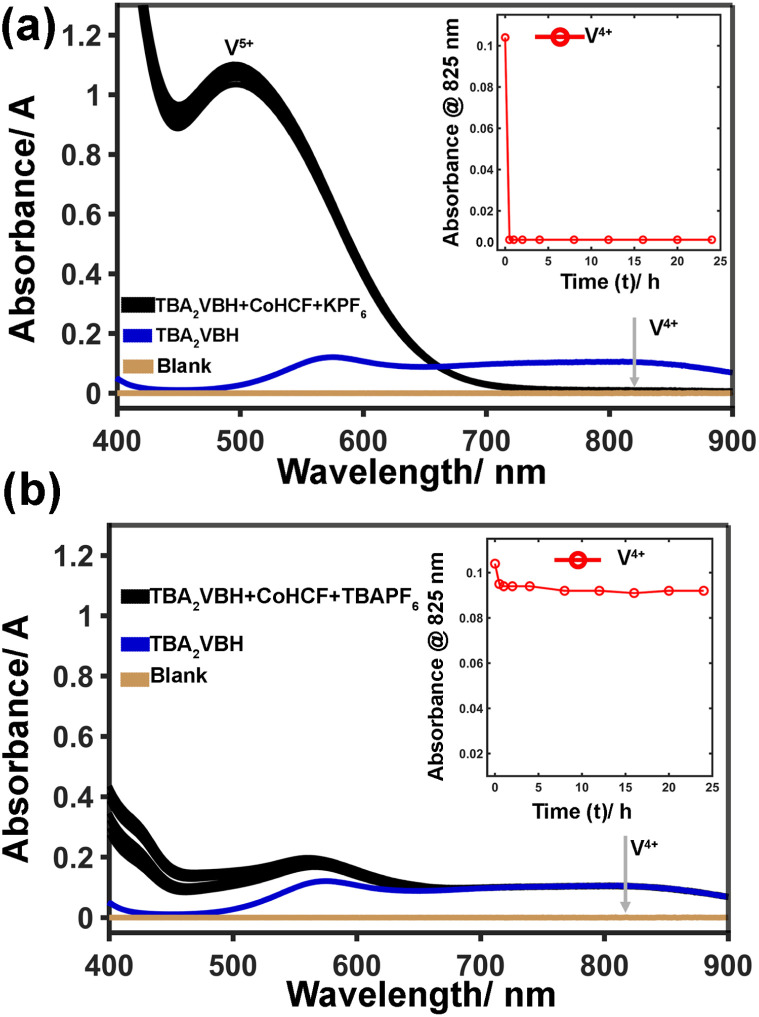
VBH^2−/1−^ Oxidation. UV-vis spectra of VBH^2−^ after addition of full equivalent of CoHCF and KPF6 done for the duration of 0.5 h, 1 h, 2 h, 4 h, 8 h, 12 h, 16 h, 20 h and, 24 h (a). UV-vis spectra of VBH^2−^ after addition of full equivalent of CoHCF and TBAPF6 done for the duration of 0.5 h, 1 h, 2 h, 4 h, 8 h, 12 h, 16 h, 20 h and, 24 h (b).

Furthermore, the time course experiments indicate that, upon mixing CoHCF(Fe^III^Co^II^), and TBA_2_VBH in the presence of an equivalent of KPF_6_, quantitative oxidation of VBH^2−^ occurs prior in the first time-point (see Fig. 5a) evidenced by a complete extinction of absorbance at 825 nm (see Fig S1a and f). In contrast, in the absence of KPF_6_ (see Fig. 5b), absorbance drops slightly in the first time-point and is constant for the subsequent 24 hours precluding further oxidation over that time period.

**Fig. 6:**
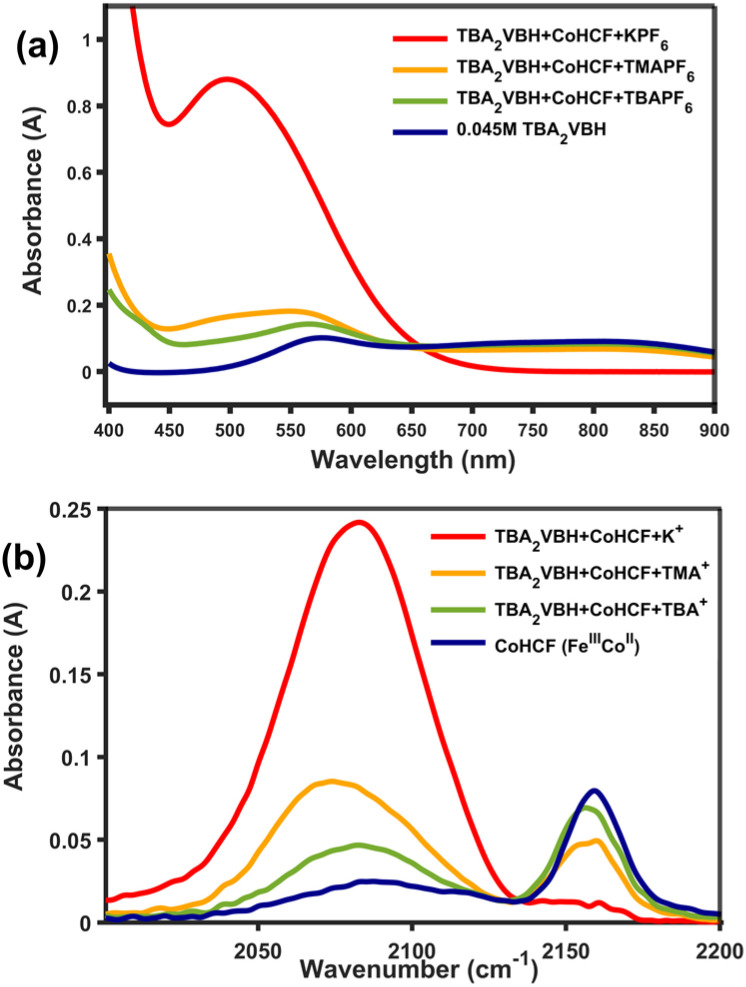
UV-vis spectra of VBH^2−^ initially and after addition of CoHCF and 1 equivalent of TBAPF_6_, TMAPF_6_ and KPF_6_ (a). Corresponding IR spectra of CoHCF (b).

To gain further insight into the possibility of tuning the indirect electrochemical reaction by adjusting electrolyte composition, TMA was also investigated as a supporting cation. The data presented in Fig. 6 and Table 1 indicate that, in the presence of equimolar TMA, oxidation of the RM proceeds to an extent that is intermediate to that of TBA and K^+^ cations. The percentage oxidation of RM was analyzed from the comparison of the experimental and simulated spectra of VBH^2−/1−^ (see Fig S2). Two effects may explain this observation. First, the smaller volume of TMA, compared to TBA, may allow some intercalation within the CoHCF lattice, but not to the extent of K^+^ (see Fig. 2c). Alternatively, the smaller size of TMA,^49^ may allow for more ions to adsorb on the surface of CoHCF, accommodating more charge. Further investigations to distinguish these possibilities, using a higher ratio of SAM to RM and by modifying SAM surface to volume, ratio *via* particle size, are underway.

### 2.2 Cyclic voltammetry of CoHCF in the presence of cations of various size

**Fig. 7:**
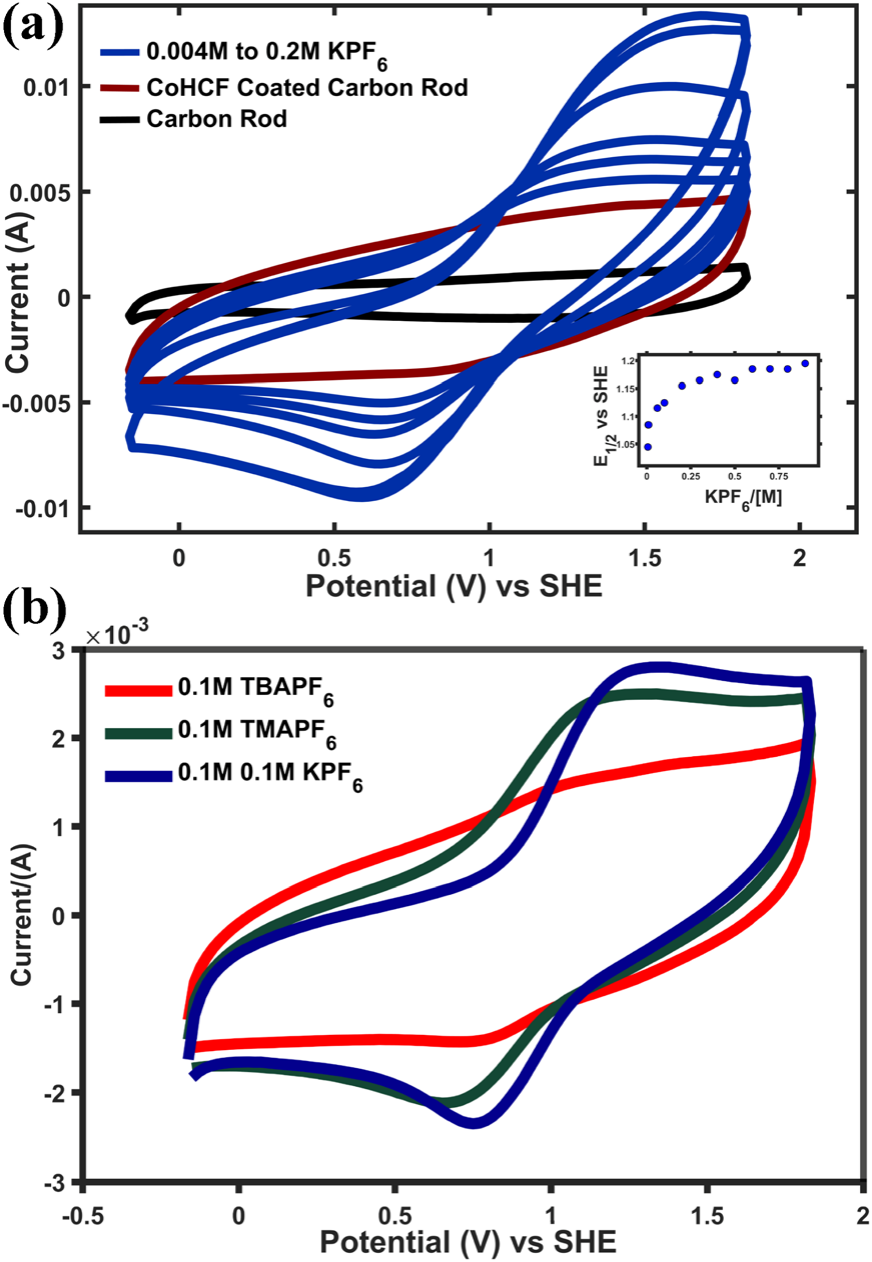
CVs of CoHCF films in MeCN, with increasing concentrations of KPF_6_ (a) (inset: shift in *E*°′ with increasing KPF_6_ concentration). CVs of CoHCF films in the presence of TBAPF_6_, TMAPF_6_ and KPF_6_ (b).

Redox intercalation of CoHCF was also investigated *via* cyclic voltammetry (CV) of a film, comprising the SAM, a PVDF dispersant and carbon nanotubes, incorporated to improve performance.^27^ While not useful to directly probe the heterogeneous reaction between RM, in solution, and colloidal SAM particles, this analysis has been widely employed to analyze MHCMs that have been electrodeposited^51^ or immobilized as particles^27,37^ on electrodes. Comparisons between measurements of films that have been electrochemically deposited^51–53^ and those that have been prepared by immobilizing particles on substrates should be made carefully since these two preparative methodologies lead to different electrode structures. In the current work the latter strategy was used to complement the solution-phase studies above and are a convenient means of screening MHCMs and studying their electrochemistry separately from RMs. A voltammogram of a CoHCF electrode collected between −0.1 and 1.8 V, in the presence of 0.2 M TBAPF_6_ in acetonitrile is mostly featureless, exhibiting only capacitive current, compared to a blank carbon rod (see Fig. 7a). Gradual addition of aliquots of KPF_6_ supporting electrolyte results in an increase of faradaic current during cycling, centered near 1 V *vs.* SHE. A well-defined peak attributable to reduction/oxidation of the low-spin Fe center of CoHCF^53^ increases sharply in current as the KPF_6_ concentration increases from 0 to 0.1 M and plateaus at higher concentration. Notably, *E*°′ also shifts cathodically more than 100 mV (see Fig. 7a, inset). To gain a further insight on reversibility and stability, a separate CV measurement of CoHCF (see Fig S3a and b) in 0.2 M KPF_6_ and 0.2 M TBAPF_6_ was performed at a scan rate of 50 mV s^−1^. The stable peak currents with no observable peak shift over 100 cycles, further confirmed the material's electrochemical and structural stability. In general agreement with solution-phase studies, Fig. 7b indicates that redox intercalation occurs in the presence of TMA, in contrast to TBA, but to a lesser extent than K^+^ and at a potential that is anodically shifted. Taken together, these effects illustrate how electrolyte composition may be tuned to optimize the dynamics of the indirect electrochemical reaction that occurs in RMFB tanks, separate from the processes occurring during C/D in the electrochemical cell. The thermodynamic framework describing this reaction, which accounts for contributions from the chemical potential of the oxidized and reduced MHCM as well as the solvation energy of the intercalation cation, is the subject of current investigation,^44^ with preliminary theoretical considerations based on DFT and periodic-DFT calculations^40^ discussed below.

### 2.3 Investigation of the effect of cations on the electrochemical kinetics of VBH

**Fig. 8:**
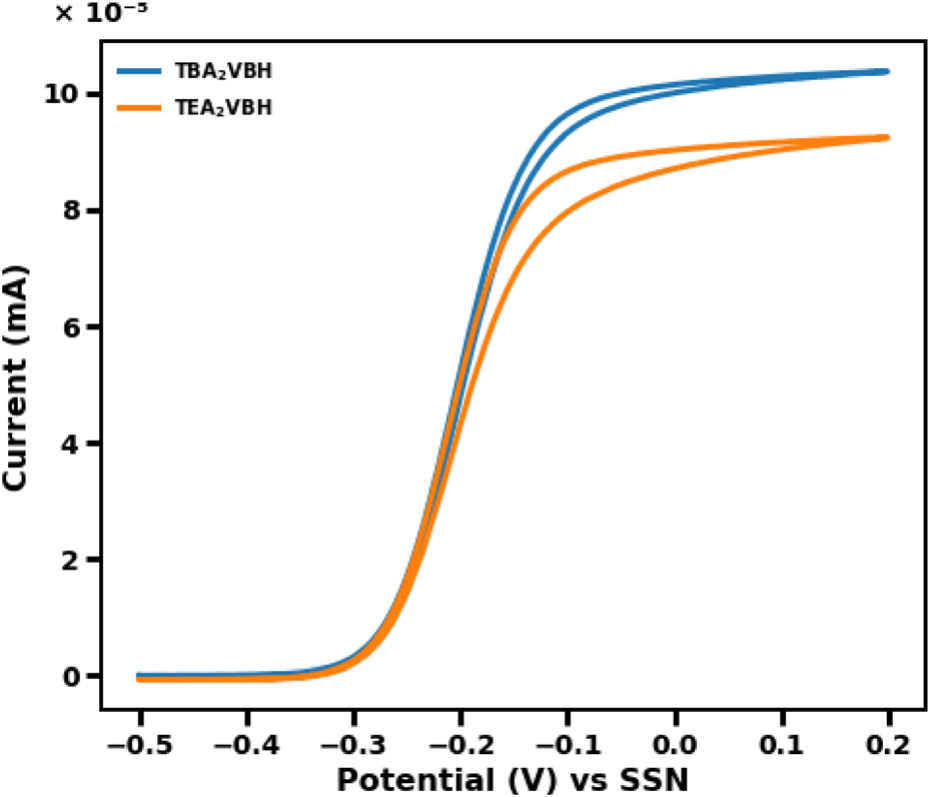
UME voltammograms of 50 mM TBA_2_VBH and 50 mM TEA_2_VBH MeCN solutions 0.01 M Ag/AgNO_3_ (0.01 M) reference, with 0.1 M TBAPF_6_ supporting electrolyte.

While the redox properties of the indirect electrochemical reaction may be fine-tuned through cation modification, such changes could influence the performance of the RM during C/D of the system. To probe these effects, ultramicroelectrode (UME) voltammetry offers a sensitive diagnostic approach, as the small surface area of the working electrode leads to very low currents, minimizing ohmic drop, and enabling rapid attainment of steady-state conditions.^54^ This setup allows for straightforward calculation of the diffusion coefficient from the limiting current, given that both the electrode size and RM concentration are controlled.^54,55^ In a direct comparison using 50 mM solutions of TBA_2_VBH and TEA_2_VBH with an 11 µm UME, the higher steady-state current observed for TEA_2_VBH indicates a larger diffusion coefficient relative to TBA_2_VBH (see Fig. 8). Furthermore, the initial slope of the steady-state voltammogram, which is often indicative of electron transfer kinetics, is similar for both mediators, suggesting comparable kinetic behavior.^54^ However, the presence of hysteresis in the steady-state voltammogram is more pronounced in the case of TEA_2_VBH suggesting that true steady-state is not achieved at all potentials, likely due to slow surface or kinetic processes, or experimental artifacts such as adsorption phenomena.^54^ Thus, while cation modification can enhance certain redox properties, it may also introduce complexities in mediator dynamics that are readily revealed through careful UME voltammetry analysis.^55^

When comparing the kinetics of redox mediators with different cation sizes, it is notable that the analogue with the larger cation often demonstrates faster kinetics.^55^ This observation may seem counterintuitive, as larger cations might be expected to hinder diffusion; however, the charge distribution of both ions and their interactions with solvent molecules all impact reorganization energy during outer sphere electron transfer.^54^ It may be that the enhanced solubility of TBA_2_VBH compared to TEA_2_VBH, and reduced tendencies for aggregation or dimerization due to steric and electrostatic effects are imparted by the bulky cation. For instance, studies on vanadium-bis-hydroxyiminodiacetate (VBH) derivatives have shown that incorporating larger tetraalkylammonium improves the solubility of the redox mediator.^11^

While larger supporting electrolyte cations are often associated with reduced diffusivity, the UME measurements presented here indicate that TBA_2_VBH exhibits a higher steady-state current and reduced hysteresis compared to TEA_2_VBH. Importantly, the initial rise (“take-off”) slopes of the voltammograms are comparable for both mediators, suggesting that their intrinsic electron-transfer kinetics are similar. The observed differences in hysteresis are therefore not interpreted as definitive evidence of faster heterogeneous kinetics for TBA_2_VBH, but rather differences in interfacial or transport-coupled behavior. Although prior studies^56^ have suggested that changes in ion pairing, solvation structure, or reorganization energy may influence electrochemical response, the data presented here do not allow these effects to be quantitatively isolated. Accordingly, reorganization energy is discussed only as a possible contributing factor and not as a definitive mechanistic explanation for the observed behavior.

### 2.4 Theoretical framework describing cation intercalation in SAMs

**Fig. 9:**
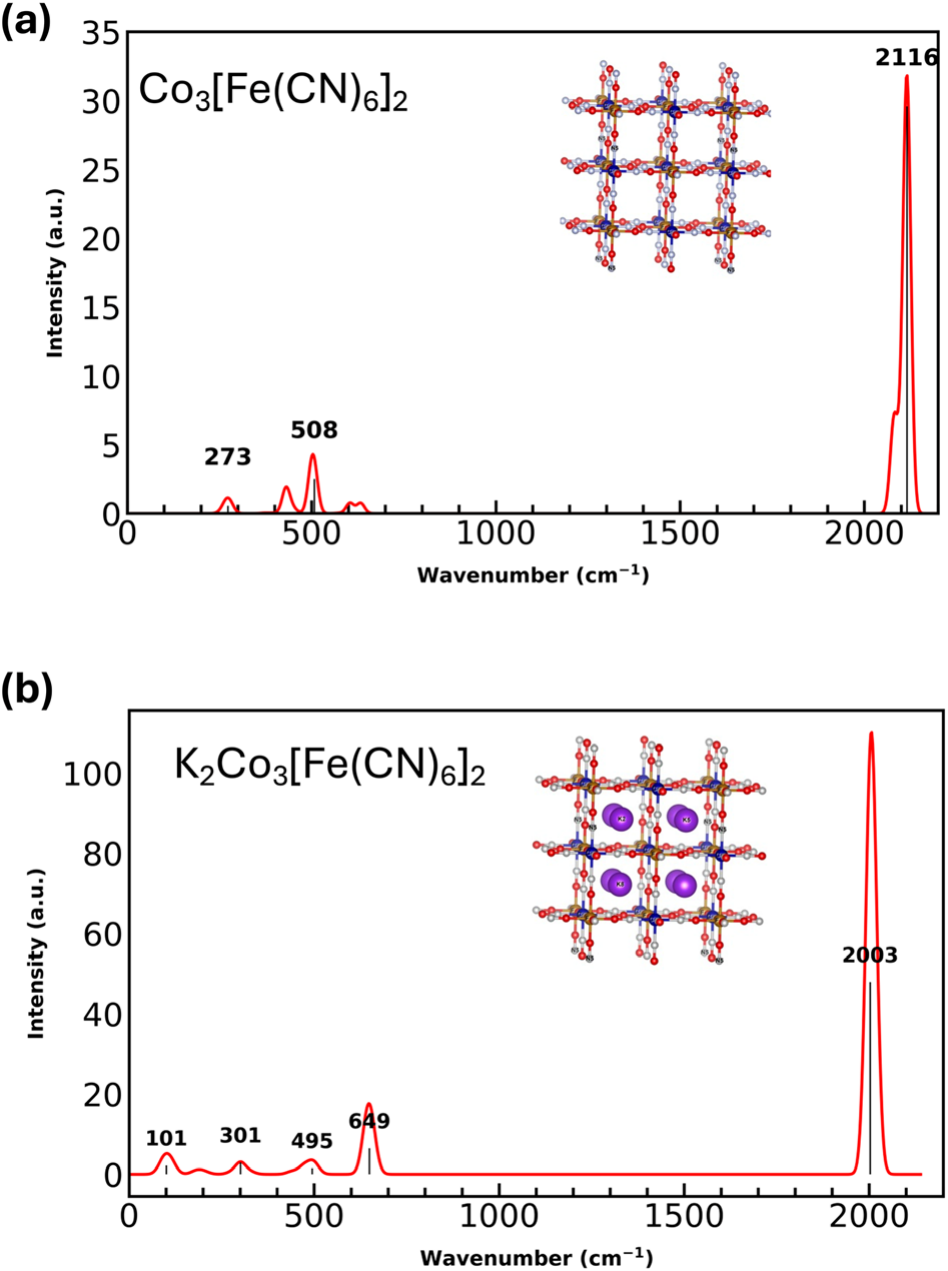
Simulated IR spectra and optimized structures of Co_3_[Fe(CN)_6_]_2_ (a) and K_2_Co_3_[Fe(CN)_6_]_2_ (b).

The simulated IR spectra of Co_3_[Fe(CN)_6_]_2_ and K_2_Co_3_[Fe(CN)_6_]_2_ (see Fig. 9) can be used to directly support interpretation of the experimental IR data (Fig. 3b and 6b) in which *ν*_CN_ serves as a diagnostic marker for CoHCF reduction. Distinct spectral regions correspond to specific bonding interactions within the lattice. For pristine CoHCF (Fe^III^Co^II^), the most prominent *ν*_CN_ feature appears at 2116 cm^−1^. Although this value is slightly red-shifted relative to experimental results above (2160 cm^−1^) and prior reports for Co^2+^/Fe^3+^ systems (2155 cm^−1^),^53^ the calculation reproduces the correct spectral region and trend, supporting the reliability of the computational model. With potassium intercalation and reduction of Fe^III^ to Fe^II^, modeled as K_2_Co_3_[Fe(CN)_6_]_2_, spectral shifts and new vibrational modes are observed (see Fig. 7b). Most notably, *ν*_CN_ redshifted from 2116 cm^−1^ to 2003 cm^−1^, mirroring the experimentally observed shift to lower frequency upon reduction.^53^ This behavior reflects increased electron density at the Fe center following reduction, which weakens Fe–C–N bonding, and lowers the stretching frequency.

In addition to *ν*_CN_, the calculated spectrum of CoHCF (Fe^III^Co^II^) exhibits Fe–C–N bending modes at 508 cm^−1^, and Co–N–C bending modes at 372 cm^−1^ and 273 cm^−1^, reflecting the local metal–ligand coordination environment. On the other hand, for K_2_Co_3_[Fe(CN)_6_]_2_, the Fe–C–N bending mode shifts from 508 to 649 cm^−1^, indicating a blue shift consistent with local lattice distortions near the intercalation sites. A new low-frequency mode below 150 cm^−1^ also appear in the intercalated structure and are assigned to vibrational motion of interstitial K^+^ ions, absent in the pure Co_3_[Fe(CN)_6_]_2_. In addition to these frequency shifts, an enhancement of vibrational intensity is observed, particularly in the high-frequency *ν*_CN_ region, likely arising from increased dipole activity due to interactions among K^+^ ions, Fe centers, and cyanide ligands, further highlighting the redox-active nature of the CoHCF host framework.

Beyond alkali metal intercalation, we are currently investigating the incorporation of larger organic cations, TMA and TBA^+^, into CoHCF. The narrow interstitial voids of the framework impose steric constraints that strongly influence intercalation behavior. Due to its smaller size, TMA is more likely to intercalate or engage in weak electrostatic interactions within the lattice. In contrast, the bulkier TBA is expected to be sterically excluded from the interior and may instead adsorb on the surface unless solvent-mediated processes or structural distortions facilitate entry. These size-dependent dynamics are under active investigation to better understand the limits of structural flexibility and guest accommodation in CoHCF and MHCM compounds. Calculating the intercalation potential requires evaluating the energy difference between the fully cation-intercalated state and the deintercalated (cation-free) state of the material. This involves determining the solvation energy and chemical potential of both the oxidized and reduced forms of the host framework, with and without the intercalated cations (*e.g.*, K^+^). While this analysis is essential for a complete thermodynamic description, it falls outside the scope of the present study and will be addressed in future work.

## 3 Conclusions

RMFBs are a promising strategy to overcome remaining RFB challenges, enabling lower-concentration solution-phase RFB active-materials, while greatly improving energy density. In this way they also facilitate mixed electrolytes, which have shown excellent performance at moderate concentration and eliminating the problem of membrane-crossover which is a major stumbling block for current generations of RFB systems.^8^ In mixed electrolytes, the concentrations required for economic viability are even more challenging since both posolyte and negolyte are present in both cells. An RMFB constructed with a mixed electrolyte would allow optimization of flow battery performance, circumventing the challenges associating with high concentration electrolyte, since nearly all the capacity would reside in the two SAMs.

In this work, we demonstrate a strategy to directly probe the heterogenous reaction between RM and SAM that occurs in RMFB rather than inferring these dynamics based on reactions occurring in the electrochemical cell. This direct measurement of SAM state-of-charge provides an additional tool to characterize and improve RMFB performance. We also report a periodic-DFT investigation of the vibrational spectroscopy of CoHCF, laying the groundwork for a thermodynamic framework that will be used to better understand the RMFB indirect electrochemical reaction.

## 4 Experimental and computational section

### 4.1 General

Zinc acetate dihydrate, ACS, 98.0–101.0%, chloroacetic acid, 99%, vanadium pentoxide (V_2_O_5_,99%), potassium hexacyanoferrate(III), ACS, (K_3_Fe(CN)_6_, 99.0%), cobalt(ii) nitrate hexahydrate (Co(NO_3_)_2_·6H_2_O, 97%), calcium chloride dihydrate (CaCl_2_·2H_2_O, 99.0–105.0%), potassium hexafluorophosphate (KPF_6_, 98%), and tetrahydrofuran (THF, 99%) were purchased from Beantown Chemical. Hydroxylamine hydrochloride, 99+% was purchased from Thermo scientific. Tetramethylammonium hexafluorophosphate (TMPF_6_, 98%), and tetrabutylammonium-fluoride trihydrate (TBA-F.3H_2_O, 95%) were bought from Ambeed. Tetrabutylammonium chloride (TBACl) was bought from CHEM-IMPEX. Dichloromethane (DCM) was purchased from VWR Chemicals. Zinc(ii)hydroxyiminodiacetate (ZnHIDA) was synthesized following the procedure outlined by Pahari *et al.*^11^

### 4.2 Physical methods

Infrared spectra were recorded on a Thermo Scientific Nicolet iS5 equipped with iD7ATR module and a diamond crystal. UV-vis spectra were collected in the Evolution 220 UV-visible Spectrophotometer (Thermo Scientific) using a quartz cuvette of 1 cm path length. Cyclic Voltammograms were recorded using Ametek VersaSTAT3 (Princeton Applied Research) potentiostat. SEM images were collected using a Hitachi SU5000 SEM with an acceleration voltage of 25 kV. PXRD powder pattern was recorded from Rigaku SmartLab X-ray diffractometer (XRD) using Cu Kα source.

### 4.3 Tetrabutylammonium vanadium(iv) bis-hydroxyiminodiacetate (TBA_2_VBH)

The RM TBA_2_VBH was synthesized according to the procedure outlined by Pahari *et al.* with some modification.^11^ The CaVBH as precursor reagent was synthesized by the addition of ZnHIDA (54.002 g, 0.25 mol), V_2_O_5_ (11.58 g, 0.063 mol), and CaCl_2_·2H_2_O (18.725 g, 0.12 mol) in 375 mL of 1 M HCl stirring at 800 rpm for 72 hours until full dissolution with conversion of yellow color to the dark blue color. CaVBH was precipitated from the dark blue solution by addition of 1200 mL of isopropanol with stirring at 800 rpm for two hours. The product was vacuum filtered, washed with 20 mL of isopropanol followed by 25 mL of acetone and dried *in vacuo* for 24 hours. (Yield: 44.10 g, 0.093 mol, 73%). IR(*ν*/cm^−1^): 3549 w, 3341 w, 2980 w, 1596 s (C=O). CaVBH (9.718 g, 0.020 mol) was added with TBAF·3H_2_O (12.92 g, 0.040 mol) in 40 mL THF, stirred for one hour and centrifuged. The CaF_2_ precipitate was discarded, and the blue supernatant was dried *in vacuo* to obtain product. (yield: 12.061 g, 0.0145 mol, 72.92%).

### 4.4 Tetrabutylammonium hexacyanoferrate(iii) (TBA_3_Fe(CN)_6_)

The TBA_3_Fe(CN)_6_ powder used as the precursor for synthesis of alkali-metal-ion-free MHCM was synthesized following the literature.^57^ TBACl (8.3520 g, 0.03 mol) was dissolved in 50 mL DCM and was mixed with 50 mL of aqueous solution of K_3_Fe(CN)_6_ (3.3105 g, 0.01 mol). The mixture was stirred for 2 hours after which the organic layer was collected and dried *in vacuo* to obtain yellow product. The product was stirred for several hours in 30 mL diethyl ether, boiled and hot filtered. The process was repeated four times and the product obtained was dried in oven at 100 °C. (yield: 6.2459 g, 0.006649 mol, 66.377%). IR(*ν*/cm^−1^): 2095 *ν*(CN), 2873 and 2959 *ν*(CH).^57^

### 4.5 Cobalt(ii) hexacyanoferrate(iii) (CoHCF)

The SAM, CoHCF was synthesized by dropwise addition using 3 : 2 ratio of Co(NO_3_)_2_·6H_2_O (5.458 g, 0.01875 mol) in 75 mL deionized water to TBA_3_Fe(CN)_6_ (11.753 g, 0.0125 mol) in 50 mL de-ionized water, with overnight stirring for 12 hours at 800 rpm. The brown precipitate was centrifuged and resuspended in water for five cycles. The product was vacuum dried at room temperature for 48 hours. (yield: 3.9775 g, 0.00662 mol). IR(*ν* or *δ*/cm^−1^): 2159 and 2087 *ν*(CN), 3640 and 3385 *ν*(OH), 1606 δ(HOH).^41,51–53^

### 4.6 PF_6_ salts

Tetrabutylammonium hexafluorophosphate (TBAPF_6_) was prepared from tetrabutylammonium bromide and potassium hexafluorophosphate in aqueous medium and was recrystallized from ethanol four times. Tetramethylammonium hexafluorophosphate (TMAPF_6_) and KPF_6_ salts were purchased from commercial sources and used after recrystallization from boiling water.^58^

### 4.7 Electrochemical details

The composite working electrode was prepared by coating the slurry of CoHCF, MWCNT, and PVDF in the ratio of 6 : 1 : 3 in NMP in a carbon rod and drying in vacuum oven for 12 hours at 75 °C. The Nonaqueous reference electrode was fabricated by submerging a piece of silver wire in 30 mM silver nitrate solution in acetonitrile with 0.2 M TBAPF6 in a glass tube fitted with vycor frit on one end (0.636 V *versus* SHE) and carbon rod was used as counter electrode. The scan rate was set at 50 mV s^−1^, with a voltage window from −0.8 V to 1.2 V *vs.* Ag/AgNO_3_ using different concentration of PF_6_ salts. The non-aqueous reference electrode were calibrated frequently using ferrocene as internal standard using 5 mM Ferrocene with 0.2 M TBAPF_6_ as supporting electrolyte.

The software program Marvin (ChemAxon)^59^ was used to draw and display chemical structures.

### 4.8 Computational details

To investigate the vibrational properties of Co_3_[Fe(CN)_6_]_2_ and K_2_Co_3_[Fe(CN)_6_]_2._ IR spectra were simulated using density functional theory (DFT) as implemented in Quantum ESPRESSO v7.2.^60^ The initial structure geometry of CoHCF was adopted from a previously published work^61^ with a cubic crystal symmetry. Prior to further calculations, a parameterization test was conducted to determine optimal values for the kinetic energy and charge density cutoffs. The structure was then fully optimized using these converged settings. Potassium ions (K^+^) were inserted into the 8c Wyckoff position of the CoHCF lattice to model the reduced, intercalated state, mimicking experimental electrochemical cycling in which K^+^ ions occupy interstitial sites to maintain electroneutrality during the reduction of low-spin Fe^3+^ centers All calculations employed a plane-wave basis set and norm-conserving pseudopotentials.^62^ The Perdew–Zunger local density approximation^61^ was used for the exchange-correlation functional. A kinetic energy cutoff of 100 Ry (1360 eV) and a charge density cutoff of 800 Ry (10 880 eV) were applied, based on prior convergence tests for CoHCF. Geometry optimizations were conducted with convergence criteria of 1 × 10^−6^ Ry for total energy and 1 × 10^−4^ Ry/Bohr for atomic forces. Following optimization, a self-consistent field calculation was used to obtain the ground-state charge density for phonon calculations. Vibrational frequencies were derived by solving the dynamical matrix.

## Author contributions

The manuscript was written through contributions of all authors. All authors have given approval to the final version of the manuscript.

## Conflicts of interest

There are no conflicts to declare.

## Supplementary Material

RA-016-D5RA08926C-s001

## Data Availability

The data associated with this manuscript, and its supplementary information (SI). Additional raw datasets generated and analyzed during the current study will be made available by the corresponding author upon reasonable request. Supplementary information is available. See DOI: https://doi.org/10.1039/d5ra08926c.
