# Supplementary material for: Fine-tuning the indirect electrochemical reaction in redox-mediated flow batteries
Source: RSC Adv. 2026 Feb 5;16(8):7344–54. doi: 10.1039/d5ra08926c (PMC12873652; doi:10.1039/d5ra08926c)
Supplement: RA-016-D5RA08926C-s001 [file RA-016-D5RA08926C-s001.pdf]

Supplementary Information (SI) for RSC Advances.
This journal is © The Royal Society of Chemistry 2026

Supplementary Information

Fine-Tuning the Indirect Electrochemical Reaction in Redox-Mediated Flow Batteries

Tulsi M. Poudel,[1] Daphne E. Poirier,[1] Marybeth Hope T. Banda,[1] Eylul Ergun,[2] Daniel Rourke,[2] Kayode O. Ojo,[1] Ertan Agar,[2] Maricris L. Mayes,[1] Patrick J. Cappillino,[1]*

AUTHOR ADDRESS

1. Department of Chemistry and Biochemistry, University of Massachusetts Dartmouth, Dartmouth, Massachusetts 02747, United States

2. Mechanical Engineering Department, Energy Engineering Graduate Program, University of Massachusetts Lowell, Lowell, Massachusetts 01854, United States

Corresponding Author

*Patrick J. Cappillino
Department of Chemistry and Biochemistry
University of Massachusetts Dartmouth
Dartmouth, MA 02747

Section 1: Experimental and Simulated UV-VIS spectra of VBH^{2-} oxidation

The UV-VIS spectra of the change in the oxidation state of the VBH^{2-} spectra was taken by measuring the change in epsilon value of each wavelength of the VBH^{2-} upon addition of the equivalent amount of the CoHCF and gradual addition of K^+ ion as KPF_6 salt from 0, 0.25, 0.5, 0.75 and 1 equivalent (see Fig. S1). For VBH^{2-} , ε(825 nm)=25.0mol^{-1} cm^{-1}, and that for VBH^{-1} , ε(485nm)=245mol^{-1} cm^{-1} was used.[1]

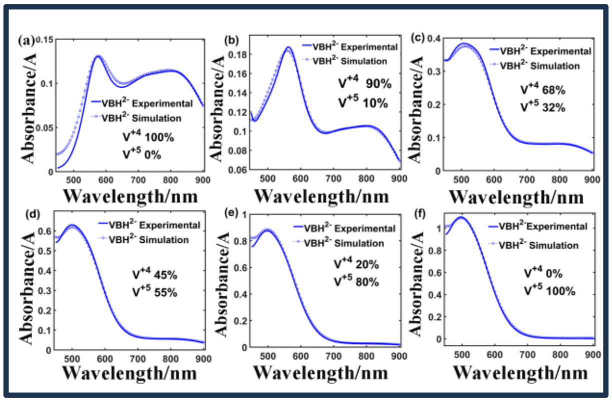

Figure S1. $VBH^{2-}/^{1-}$ Oxidation. **(a)**. UV-vis spectra of VBH^{2-} **(b)**. After addition of full equivalent of CoHCF with no KPF_6. **(c).** 0.25 KPF_6 **(d).** 0.5 KPF_6 **(e).** 0.75 KPF_6 **(f).** 1 KPF_6

Similarly, the UV-VIS spectra of the change in the oxidation state of the VBH^{2-} was taken by measuring the change in epsilon value of each wavelength of the VBH^{2-} upon addition of the same equivalent of the CoHCF and the cations (TBA^+, TMA^+, & K^+) corresponding to its PF_6 salts (see Fig. S2)

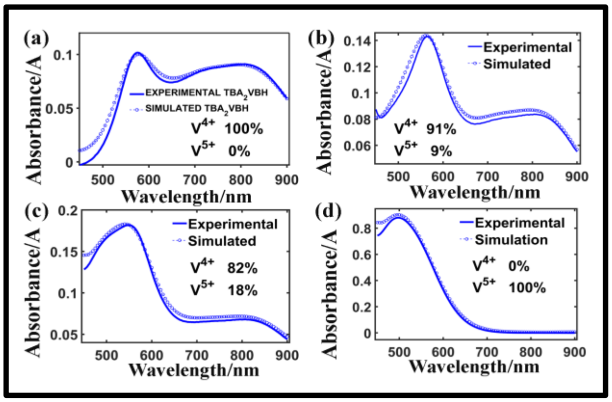

Figure S2. $VBH^{2-}/^{1-}$ Oxidation. **(a).** UV-vis spectra of VBH^{2-} **(b)**. After addition of full equivalent of CoHCF and $TBAPF_6$. **(c).** Adding Full equivalents of CoHCF and $TMAPF_6$ **(d).** After addition of full equivalents of CoHCF and KPF_6

Section 2: CV Analysis of CoHCF following addition of 0.2M $TBAPF_6$+ 0.2M KPF_6 Addition in MeCN

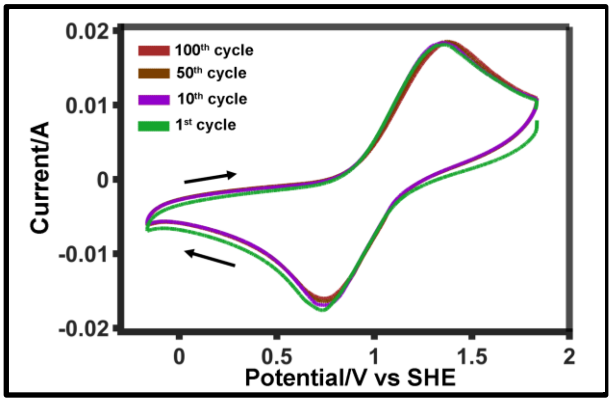

Figure S3. a) CV of CoHCF done for 100 cycles coated on carbon rod in 6:1:3 ratio of CoHCF, MWCNT, & PVDF in NMP. 0.2M $TBAPF_6$ and 0.2M KPF_6 were used as electrolyte solution in MeCN. Scan rate was 0.05 V/s.

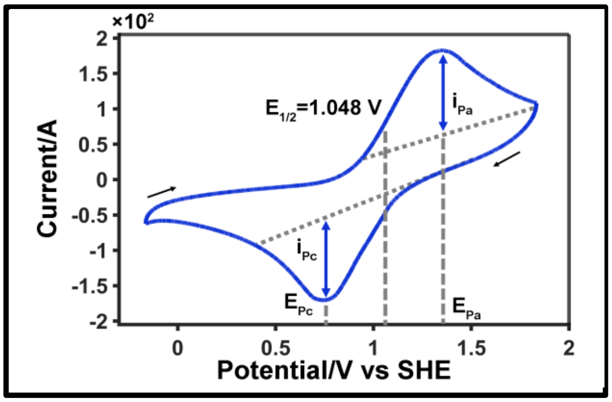

Figure S3. b) CV of CoHCF coated on carbon rod in 6:1:3 ratio of CoHCF, MWCNT, & PVDF in NMP. 0.2M $TBAPF_6$ and 0.2M KPF_6 were used as electrolyte solution in MeCN. Scan rate was 0.05 V/s. dEp value calculated as 0.60 V and the anodic to cathodic peak current ratio as 0.97.

REFERENCE

(1) Pahari, S. K.; Gokoglan, T. C.; Chaurasia, S.; Bolibok, J. N.; Golen, J. A.; Agar, E.; Cappillino, P. J. Toward High-Performance Nonaqueous Redox Flow Batteries through Electrolyte Design. *ACS Appl. Energy Mater.* **2023**, *6* (14), 7521–7534. https://doi.org/10.1021/acsaem.3c00910.
